# Reference-Driven Compressed Sensing MR Image Reconstruction Using Deep Convolutional Neural Networks without Pre-Training

**DOI:** 10.3390/s20010308

**Published:** 2020-01-06

**Authors:** Di Zhao, Feng Zhao, Yongjin Gan

**Affiliations:** 1 Key Laboratory of Complex System Optimization and Big Data Processing, Guangxi Colleges and Universities, Yulin Normal University, Yulin 537000, China; zhaodi@ylu.edu.cn; 2 School of Physics and Telecommunication Engineering, Yulin Normal University, Yulin 537000, China; yongjin_gan@ylu.edu.cn

**Keywords:** reference-driven, compressed sensing, magnetic resonance imaging, deep image prior, deep learning

## Abstract

Deep learning has proven itself to be able to reduce the scanning time of Magnetic Resonance Imaging (MRI) and to improve the image reconstruction quality since it was introduced into Compressed Sensing MRI (CS-MRI). However, the requirement of using large, high-quality, and patient-based datasets for network training procedures is always a challenge in clinical applications. In this paper, we propose a novel deep learning based compressed sensing MR image reconstruction method that does not require any pre-training procedure or training dataset, thereby largely reducing clinician dependence on patient-based datasets. The proposed method is based on the Deep Image Prior (DIP) framework and uses a high-resolution reference MR image as the input of the convolutional neural network in order to induce the structural prior in the learning procedure. This reference-driven strategy improves the efficiency and effect of network learning. We then add the *k*-space data correction step to enforce the consistency of the *k*-space data with the measurements, which further improve the image reconstruction accuracy. Experiments on in vivo MR datasets showed that the proposed method can achieve more accurate reconstruction results from undersampled *k*-space data.

## 1. Introduction

Magnetic Resonance Imaging (MRI) is an important non-invasive procedure that can provide critical structural, functional, and anatomical information about a patient. Nevertheless, the long time required for the scanning procedure may result in motion artifacts that can degrade image quantity and lead to misinterpretation of data, as well as sometimes cause discomfort for the patient. Accelerating the process of data acquisition without degrading the image reconstruction quality has always been one of the goals of MRI technology research. Compressed Sensing MRI (CS-MRI) [1,2,3,4] is an effective approach to reconstructing high-quality MR images from undersampled *k*-space data. CS-MRI utilizes the sparsity (or compressibility) of the MR image as prior information and builds the reconstruction model as the combination of the data fidelity term in *k*-space and the regularization constraint under some sparsifying operation. The available prior used in classical CS-MRI can be the sparsity in specific transform domains (e.g., gradient and wavelet) [2,5,6], as well as a more fixable sparse representation obtained from data via dictionary learning [7,8,9,10]. In addition, the structural prior information is drawing increased attention, because it can be acquired from a known high-resolution reference image [11,12,13] and introduces support information [14,15] or structural sparsity (e.g., group sparsity and block sparsity) [16,17,18] into the reconstruction model based on the union of subspaces theory [19,20].

Over the past several years, deep learning has attracted a great deal of attention in the medical imaging field, because it achieves better performance than conventional model based methods in terms of denoising, segmentation, classification, and accelerated MRI tasks [21,22,23,24,25,26,27,28,29,30,31]. Due to its ability to learn from data, deep learning based CS-MRI also shows superior image reconstruction performance. However, the network training procedure usually requires large datasets, which is a challenge in clinical applications because large, high quality, and patient-based datasets can be difficult to obtain due to patient privacy concerns.

Recently, Ulyanov et al. proposed a Deep Image Prior (DIP) framework [32], which performs very well in solving imaging inverse problems without pre-training. In DIP, no pre-training dataset is needed, a convolutional neural network (CNN) is initialized with random parameters, and only random noise is prepared as the network input. Research related to DIP has focused on natural image denoising, inpainting, super-resolution reconstruction [33,34], PET image reconstruction [35,36], and even compressed sensing recovery problems [37].

Leveraging the key concept of DIP, to overcome the difficulty of MR dataset acquisition and to improve learning efficiency, we used the DIP framework and introduced a structural prior provided by a high-resolution reference MR image with the same anatomical structure (which usually can be obtained by being fully sampled in advance) and proposed a reference-driven compressed sensing MR image reconstruction method. Our proposed method can achieve more accurate MR reconstruction than DIP. Our contributions can be summarized as follows.

(1) We propose a novel deep learning based compressed sensing MR image reconstruction method that does not require any pre-training procedure. This significantly reduces the dependence of traditional deep learning methods on datasets, which has always been a challenge in clinical applications.

(2) The proposed method utilizes high-resolution reference images as the input for CNNs, so that the structural similarity between the target and the reference MR image can be introduced as prior information into the network, which improves the efficiency of learning.

(3) The *k*-space data correction step is added to force the final reconstructed *k*-space data to be consistent with the prior measurement, which further improves the reconstruction accuracy.

The rest of this paper is organized as follows. Section 2 describes the proposed method in detail. Section 3 shows experimental results from three groups of in vivo MR scans, and data acquisition, undersampled masks, and network setup details are also included. Finally, conclusions are drawn in Section 4.

## 2. Methodology

### 2.1. Proposed Method

**Figure 1:**
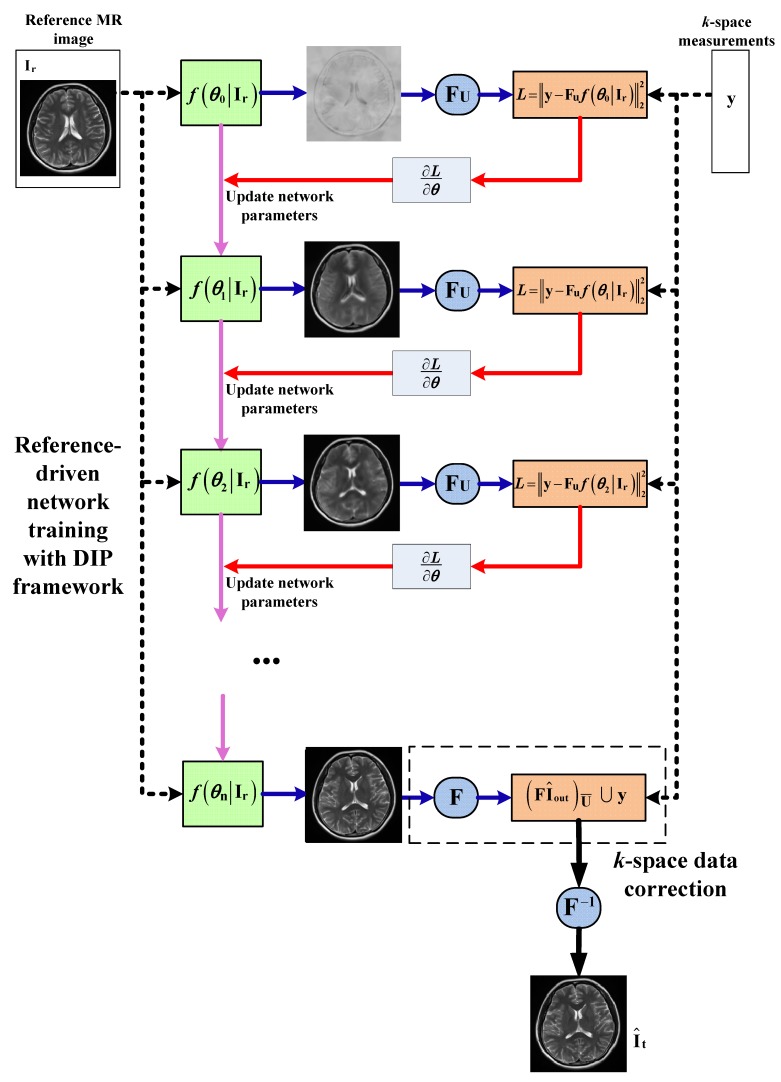
Overview of our proposed method. DIP, Deep Image Prior.

An overview of our proposed method is depicted in Figure 1. The reconstruction for the target MR image can be achieved in two steps: (1) reference-driven network training with DIP framework; and (2) data correction. In the first step, we learn the network’s parameters by solving an optimization problem and obtain the output MR image of the trained network. In the data correction step, we replace the *k*-space data of the output MR image with the original undersampled measurements and finally reconstruct the target MR image. The following sections will provide further explanation of this method.

A. Reference-driven network training with DIP framework

Let $I_{t}\in C^{N\times N}$ denote the target MR image desired to be reconstructed and $I_{r}\in C^{N\times N}$ denote a high-resolution reference MR image with similar anatomical structure to the target image acquired in advance. The proposed reference-driven network training with DIP can be formulated as the following optimization:

(1) $$\begin{array}{c} \hat{\theta }=\underset{\theta }{\mathrm{argmin}}{∥y-F_{u}f\left(\theta |I_{r}\right)∥}_{2}^{2} \end{array}$$

where $y\in C^{N\times 1}$ is the *k*-space measurements of the target MR image, $F_{u}$ denotes an undersampled Fourier transform operator, and ∥·∥ is the $l_{2}$ norm. $f(\theta |I_{r})$ is an untrained deep CNN parametrized by θ and with the fully known reference image as input. The objective function employed in Equation (1) restricts the data consistency between the CNN output and *k*-space measurements. In other words, the parameters of CNN are iteratively optimized so that the output of the network is as close to the target MR image as possible.

Then, we obtain the output ${\hat{I}}_{\mathrm{out}}$ of the trained CNN such that:

(2) $$\begin{array}{c} {\hat{I}}_{\mathrm{out}}=f\left(\hat{\theta }|I_{r}\right) \end{array}$$

With our proposed reference-driven method, the patient’s own MR image (the reference image) is utilized as the CNN input instead of as random noise. Due to the structural similarity between the target and reference MR images, this strategy efficiently introduces the structural prior to the target image to the network training procedure.

B. Data correction

Applying data correction operator Cor(·) to the output of the network ${\hat{I}}_{\mathrm{out}}$, we obtain new *k*-space data as follows:

(3) $$\begin{array}{c} y_{\mathrm{new}}=Cor\left({\hat{I}}_{\mathrm{out}}\right)={\left(F{\hat{I}}_{\mathrm{out}}\right)}_{\bar{U}}⋃y \end{array}$$

Here, F denotes the Fourier transform, y is the measurement of the target MR image collected at spatial locations corresponding to the undersampled mask U, and $\bar{U}$ denotes the complementary set of U. The *k*-space data correction operation shown in Equation (3) enforces consistency with the priori acquired measurements, so that the reconstruction error will focus on the missing *k*-space data. Experiments show that this strategy is highly effective. The final reconstruction can then be obtained through the inverse Fourier transform of $y_{\mathrm{new}}$:

(4) $$\begin{array}{c} {\hat{I}}_{t}=F^{-1}\left(y_{\mathrm{new}}\right) \end{array}$$

### 2.2. Network Architecture

**Figure 2:**
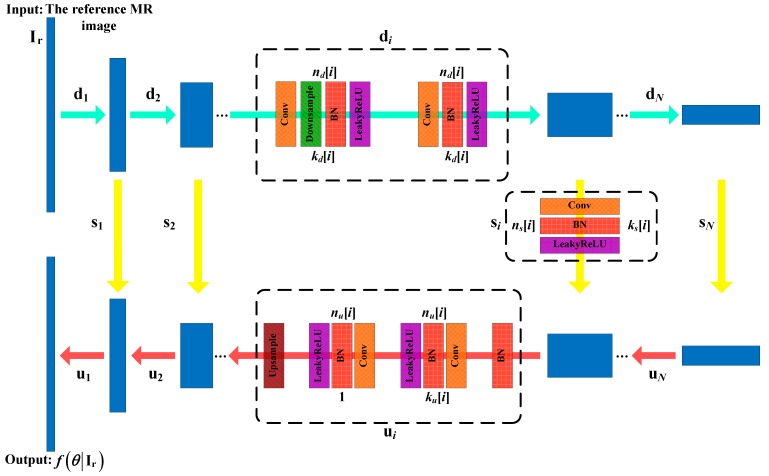
Network architecture [32] used in the proposed method.

Figure 2 depicts the CNN architecture employed in our proposed method, which is an encoder-decoder (“hourglass”) architecture with skip connections, the same as in [32]. The skip connections (marked by yellow arrows) link the encoding path (upper side) and decoding path (bottom side) and allow the integration of features from different resolutions. The network consists of repetitive applications of the convolutional (Conv) layer, batch normalization (BN) layer, leaky rectified linear unit (LeakyReLU) layer, downsampling with stride, and upsampling with bilinear interpolation. For simplicity, we denote the number of filters at depth *i* for downsampling, upsampling, and skip connections as $n_{d}\left[i\right]$, $n_{u}\left[i\right]$, and $n_{s}\left[i\right]$, respectively, and the corresponding kernel sizes are $k_{d}\left[i\right]$, $k_{u}\left[i\right]$, and $k_{s}\left[i\right]$, respectively. The variable *L* is the maximal depth of the network.

## 3. Experiments and Results

In this section, we compare our proposed method with the state-of-the-art DIP method presented in [32] to confirm the former’s better performance. To ensure a fair comparison, the same network architecture was used for both methods. In addition, to show the effectiveness in increasing reconstruction quality from highly undersampled measurements, the zero-filling image is also shown for comparison.

### 3.1. Experimental Setup

A. Data acquisition

**Figure 3:**
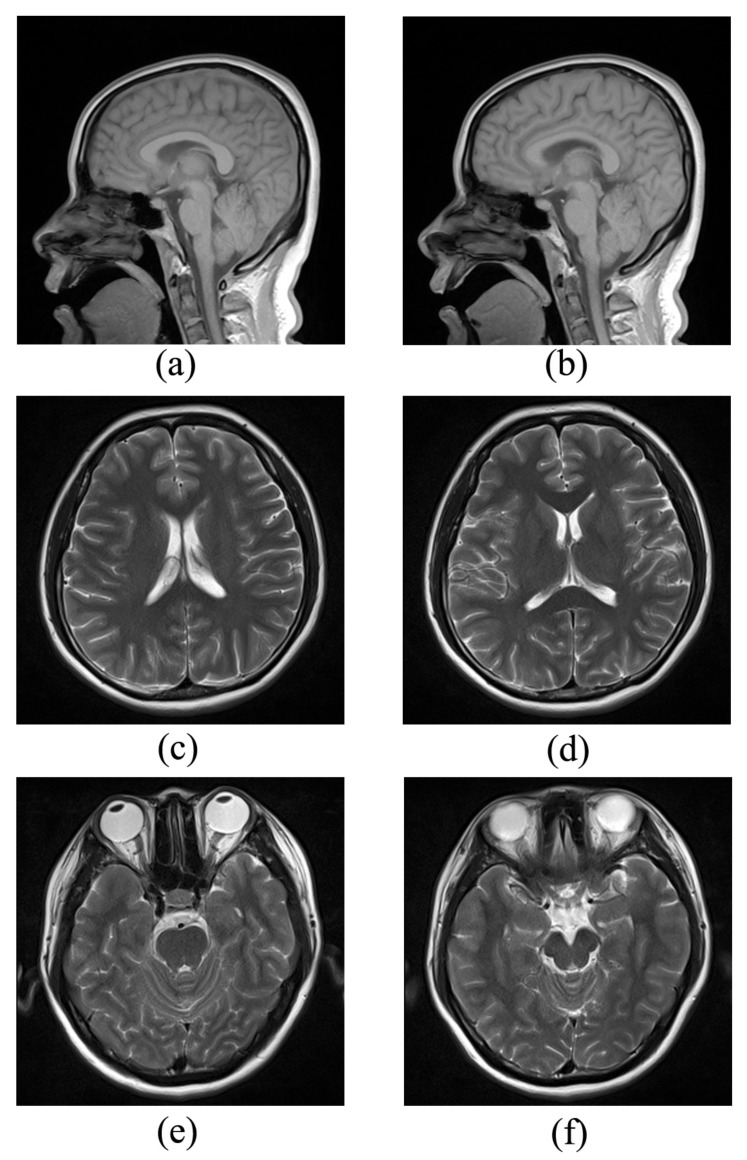
The MR images. Brain A: the reference image (**a**) and target image (**b**); Brain B: the reference image (**c**) and target image (**d**); Brain C: the reference image (**e**) and target image (**f**).

**Figure 4:**
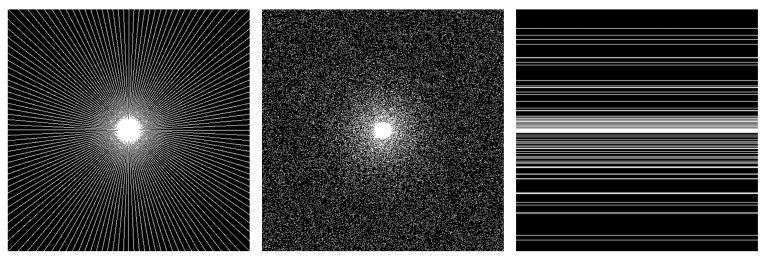
The different undersampled masks with a sampling rate of 15%. From left to right: radial mask, variable density mask, and Cartesian mask.

To demonstrate the performance of the proposed method, we performed the simulations on three groups of compressible in vivo MR images, as shown in Figure 3. To simulate the data acquisition, we undersampled the 2D discrete Fourier transform of the MR images that were from in vivo MR scans. The first group of scanned data (Brain A) was acquired from a 3T Siemens MRI scanner using the GR sequence with a flip angle of 70° and TR/TE = 250/2.5 ms. The Field Of View (FOV) was 220mm×220mm, and the slice thickness was 5.0mm. The reference and target images were of size 512×512, as shown in Figure 3a,b. The second and third groups of scanned data (Brain B and Brain C) were also acquired from the 3T Siemens scanner, but using the SE sequence (120° flip angle, TR/TE = 4000/91 ms, 176mm×176mm field of view, 5.0mm slice thickness). The MR images in Brain B and Brain C were of size 256×256 and are shown in Figure 3c–f, respectively. Three different undersampling masks were used in our experiments: a radial mask, Cartesian mask, and variable density mask. These are shown in Figure 4.

B. Network training

The network architectures were given above. The network parameters $\theta_{0}$ were initialized randomly at the first iteration. Table 1 shows the hyperparameters for the experiments conducted on Brain A, Brain B, and Brain C.

The models were implemented on the Ubuntu 16.04 LTS (64 bit) operating system, running on an Intel Core i9-7920X 2.9 GHz CPU and Nvidia GeForce GTX 1080Ti GPU (11 GB memory) in the open framework Pytorch with CUDA and CUDNN support.

C. Performance evaluation

To evaluate the quantitative performance of the proposed method, we measured the relative error, Peak Signal-to-Noise Ratio (PSNR), and Structural Similarity Index (SSIM) [38], which is more often typically used in the imaging field for consistency with human eye perception:

(5) $$\begin{array}{c} Relative\;error=\frac{\hat{x}-x}{x} \end{array}$$

(6) $$\begin{array}{c} PSNR=10\mathrm{lg}\frac{NN{\left(MAX_{x}\right)}^{2}}{\sum_{i=1}^{N}\sum_{j=1}^{N}\left[\hat{x}\left(i,j\right)-x\left(i,j\right)\right]} \end{array}$$

(7) $$\begin{array}{c} SSIM=\frac{\left(2\mu_{x}\mu_{\hat{x}}+c_{1}\right)\left(2\sigma_{x\hat{x}}+c_{2}\right)}{\left(\mu_{x}^{2}+\mu_{\hat{x}}^{2}+c_{1}\right)\left(\sigma_{x}^{2}+\sigma_{\hat{x}}^{2}+c_{2}\right)} \end{array}$$

where $\hat{x}$ and x denote the reconstructed image and the ground truth with the same size of N×N and $MAX_{x}$ is the largest value in x. Moreover, in Equation (7), $\mu_{x}$, $\mu_{\hat{x}}$, $\sigma_{x}$, and $\sigma_{\hat{x}}$ represent the means and standard deviations of x and $\hat{x}$, respectively, and $\sigma_{x\hat{x}}$ denotes the cross-covariance between x and $\hat{x}$ and constants $c_{1}=0.01$ and $c_{2}=0.03$.

### 3.2. Results

A. Reconstruction under different sampling rates

Table 2 shows the quantitative performance of our proposed method, the classic DIP method and zero-filling reconstruction on three groups of in vivo MR images at different sampling rates under the Cartesian mask. Due to the randomness involved in the training procedure (the initial network parameters for our method; both initial network parameters and network input for DIP), all results were the average values of 30 times of running. It can be seen that the proposed method achieved better performance with fewer relative errors and higher PSNRs and SSIMs (marked by red), which means that the proposed method can reconstruct the target MR image more accurately.

**Figure 5:**
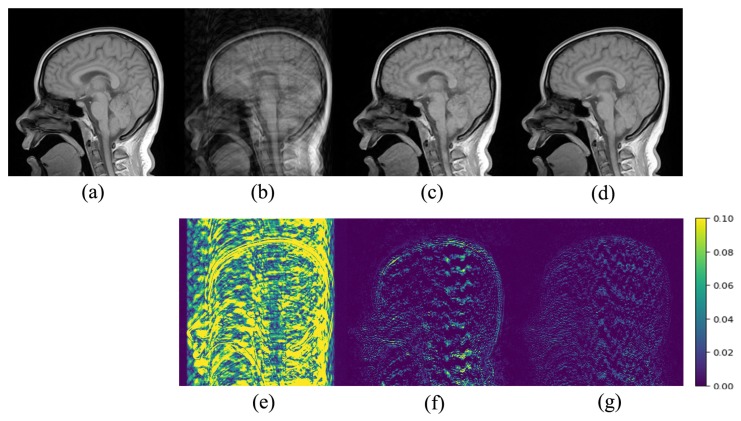
Comparison of the reconstruction results of the target MR image: (**a**) in Brain A using the Cartesian undersampled mask with 20% sampling rate; (**b**) zero-filling reconstruction; (**c**) DIP reconstruction; (**d**) the proposed method reconstruction and corresponding error maps (**e**)–(**g**).

**Figure 6:**
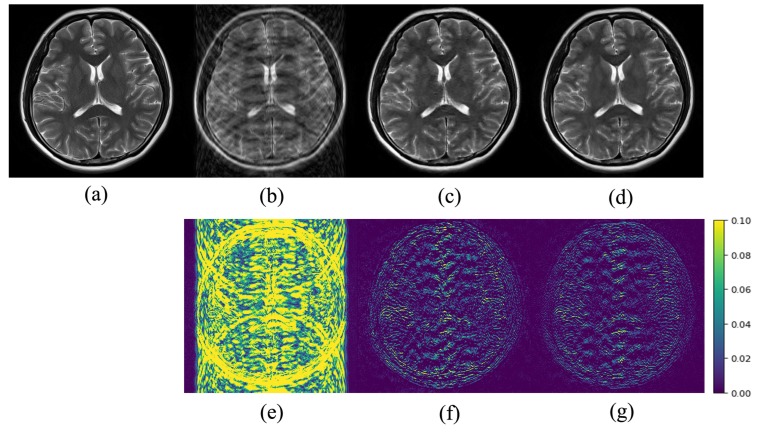
Comparison of the reconstruction results of the target MR image: (**a**) in Brain B using the Cartesian undersampled mask with 30% sampling rate; (**b**) zero-filling reconstruction; (**c**) DIP reconstruction; (**d**) the proposed method reconstruction and corresponding error maps (**e**)–(**g**).

**Figure 7:**
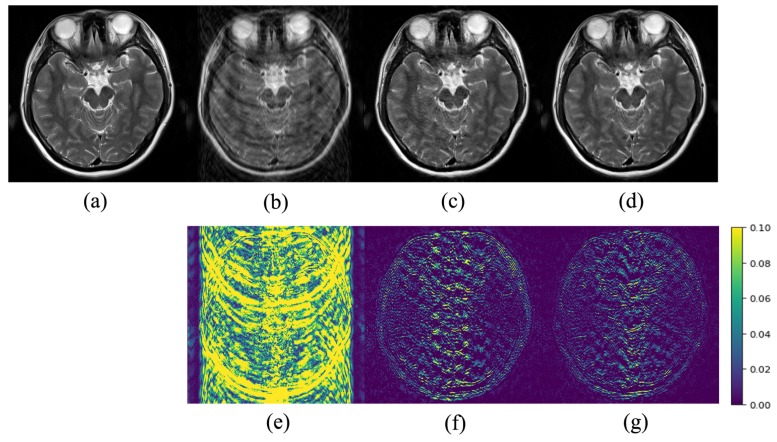
Comparison of the reconstruction results of the target MR image: (**a**) in Brain C using the Cartesian undersampled mask with 30% sampling rate; (**b**) zero-filling reconstruction; (**c**) DIP reconstruction; (**d**) the proposed method reconstruction and corresponding error maps (**e**)–(**g**).

Figure 5, Figure 6 and Figure 7 show a visual comparison of the reconstructions under Cartesian undersampling. From these figures, it is obvious that our proposed method reconstructed the higher quality image with more structural details and fewer artifacts. The corresponding error maps show that the images reconstructed by our proposed method were closer to the target image than the classic DIP method.

Table 3 shows the computational time at different sampling rates under the Cartesian mask for DIP and the proposed methods on Brain B and Brain C. Here, the computational time was the total time cost of 5000 iterations. Compared to the DIP method, our proposed method did not save time because the output of the network needed to be undersampled after each iteration so as to update the loss function. In spite of this, the significant improvement in reconstruction accuracy made the proposed method attractive.

B. Reconstruction under different undersampled masks

To further demonstrate the effectiveness of the proposed method under different undersampled masks, we also used the radial undersampled mask and variable density undersampled mask to compare the reconstructed performance. The quantitative results of three groups of MR data are presented in Table 4. It is clear that the proposed method still showed significantly improved performance under different sampling masks.

C. Convergence analysis

**Figure 8:**
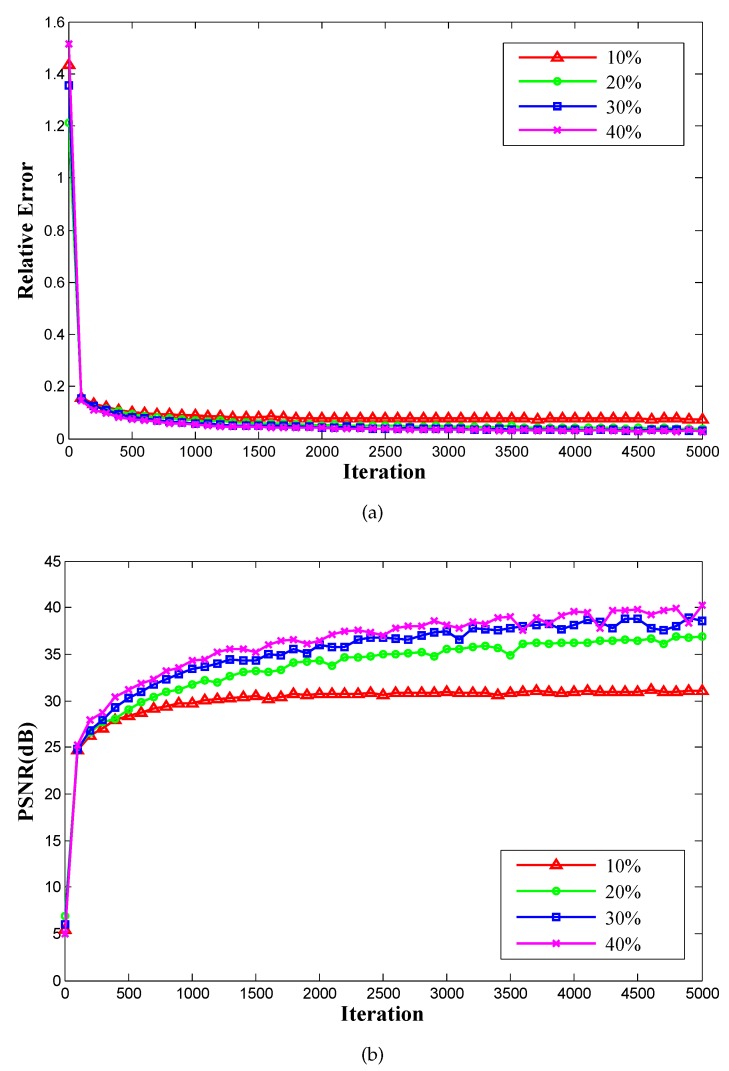
Results of the convergence for the proposed method at different sampling rates under the Cartesian undersampled mask: relative error curves (**a**) and PSNR curves (**b**).

Here, we detect the convergence of the proposed method by conducting experiments on Brain A at different sampling rates under the Cartesian undersampled mask. The curves in Figure 8a,b present the relative errors and PSNR values (average values of 30 times of running) at every 100 iterations. From the curves, we see that the proposed method gradually and stably converged to low/high values as the number of iterations increased.

D. Anti-noise performance analysis

**Figure 9:**
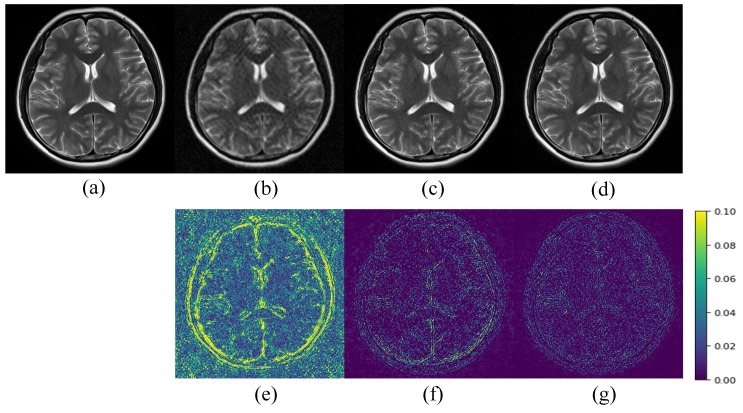
Comparison of the reconstruction results of the target MR image: (**a**) in Brain B at a 30% sampling rate under the radial undersampled mask with additive Gaussian noise; (**b**) zero-filling reconstruction; (**c**) DIP reconstruction; (**d**) the proposed method reconstruction and corresponding error maps (**e**)–(**g**).

In order to evaluate the robustness against measurement noise of the proposed method, we performed experiments on Brain B with additive Gaussian noise. Figure 9 shows the comparison of the reconstructed images under the radial undersampled mask with a 30% sampling rate. The additive Gaussian noise is complex-valued because the MRI data in *k*-space is complex-valued, with the mean μ=0 and standard deviation σ=1. The reconstructed target images by the classical DIP method and the proposed method were both acceptable, and the proposed method achieved more accurate reconstruction and fewer artifacts. The quantitative results shown in Table 5 further support the improved performance of our proposed method in the presence of measurement noise.

## 4. Conclusions

In this paper, we proposed a novel deep learning based method, which did not require patient-based training datasets, for MR image reconstruction from undersampled *k*-space data. First, our proposed method reconstructed the target MR image using the DIP framework so as to reduce the dependence of the learning on training datasets. Next, we used the known high-resolution reference MR image with a similar anatomical structure as the input of the CNN. This strategy introduced the structural information and improved the efficiency of the learning. The final *k*-space data correction step further increased the accuracy of the reconstruction by enforcing the data consistency. The experimental results demonstrated that the proposed method could successfully reconstruct the MR image without pre-training and also further improve the reconstruction quality on preserving texture details and removing artifacts compared with the conventional DIP method.

## Figures and Tables

**Table 1:** Hyperparameter setting for the experiments.

Hyperparameters		Images	
	Brain A	Brain B	Brain C
*L*	5	6	6
$n_{d}$	[8, 16, 32, 64, 128]	[6, 32, 64, 128, 128, 128]	[6, 32, 64, 128, 128, 128]
$n_{u}$	[8, 16, 32, 64, 128]	[6, 32, 64, 128, 128, 128]	[6, 32, 64, 128, 128, 128]
$n_{s}$	[8, 8, 8, 8, 8]	[4, 4, 4, 4, 4, 4]	[4, 4, 4, 4, 4, 4]
$k_{d}$	[3, 3, 3, 3, 3]	[3, 3, 3, 3, 3, 3]	[3, 3, 3, 3, 3, 3]
$k_{u}$	[3, 3, 3, 3, 3]	[3, 3, 3, 3, 3, 3]	[3, 3, 3, 3, 3, 3]
$k_{s}$	[1, 1, 1, 1, 1]	[1, 1, 1, 1, 1, 1]	[1, 1, 1, 1, 1, 1]
Number of iterations	5000	5000	5000
Learning rate	0.01	0.01	0.01

**Table 2:** Relative errors, PSNR, and SSIM values of reconstruction by different methods under the Cartesian undersampled mask.

**Images**	**Methods**		**10%**			**20%**	
		**Relative Error (%)**	**PSNR (dB)**	**SSIM**	**Relative Error (%)**	**PSNR (dB)**	**SSIM**
Brain A	Zero-filling	23.19	21.2991	0.6658	16.96	24.0131	0.7340
	DIP	17.76	23.6856	0.8169	6.59	32.4196	0.9505
	Proposed method	**7.50**	**31.1077**	**0.9443**	**3.69**	**37.2870**	**0.9793**
Brain B	Zero-filling	39.71	18.3797	0.5671	20.79	23.9985	0.7116
	DIP	34.94	19.5328	0.6738	13.86	27.5478	0.9023
	Proposed method	**19.37**	**24.6170**	**0.8443**	**9.43**	**30.8447**	**0.9516**
Brain C	Zero-filling	33.65	18.9034	0.5874	17.02	24.8250	0.7216
	DIP	31.13	19.5803	0.6770	13.28	27.0063	0.8877
	Proposed method	**20.93**	**23.0289**	**0.8027**	**10.15**	**29.3177**	**0.9317**
**Images**	**Methods**		**30%**			**40%**	
		**Relative Error (%)**	**PSNR (dB)**	**SSIM**	**Relative Error (%)**	**PSNR (dB)**	**SSIM**
Brain A	Zero-filling	5.92	33.1598	0.8215	4.27	35.9904	0.8409
	DIP	4.08	36.6919	0.9734	3.82	37.3045	0.9768
	Proposed method	**2.31**	**41.3355**	**0.9900**	**2.02**	**42.5144**	**0.9918**
Brain B	Zero-filling	20.93	23.9418	0.7185	10.70	29.7719	0.8024
	DIP	9.67	30.6624	0.9455	7.45	32.9221	0.9644
	Proposed method	**7.39**	**32.9747**	**0.9665**	**5.58**	**35.4324**	**0.9781**
Brain C	Zero-filling	16.42	25.1364	0.7408	8.94	30.4097	0.8071
	DIP	10.26	29.2226	0.9287	8.05	31.3531	0.9513
	Proposed method	**7.83**	**31.5733**	**0.9559**	**5.83**	**34.1343**	**0.9718**

**Table 3:** The computational time at different sampling rates under the Cartesian mask for DIP and the proposed methods.

Images	Methods	Computational Time			
		10%	20%	30%	40%
Brain B	DIP	3 m 12 s	3 m 9 s	3 m 14 s	3 m 4 s
	Proposed method	3 m 21 s	3 m 8 s	3 m 14 s	3 m 12 s
Brain C	DIP	3 m 7 s	3 m 19 s	3 m 9 s	3 m 8 s
	Proposed method	3 m 14 s	3 m 19 s	3 m 6 s	3 m 16 s

**Table 4:** Relative errors, PSNR, and SSIM values of reconstruction by different methods at 20% sampling rates under the radial undersampled mask and variable density undersampled mask.

Images	Methods	Radial Undersampled Mask (20%)			Variable Density Undersampled Mask (20%)		
		Relative Error (%)	PSNR (dB)	SSIM	Relative Error (%)	PSNR (dB)	SSIM
Brain A	Zero-filling	6.03	33.0053	0.8902	8.61	29.9079	0.8346
	DIP	3.98	36.8254	0.9754	5.08	34.6761	0.9638
	Proposed method	**2.23**	**41.6545**	**0.9897**	**2.93**	**39.2628**	**0.9830**
Brain B	Zero-filling	17.61	25.4440	0.7424	22.49	23.3150	0.6674
	DIP	9.43	30.8724	0.9492	11.09	29.4899	0.9250
	Proposed method	**3.98**	**36.8254**	**0.9754**	**5.08**	**34.6761**	**0.9638**
Brain C	Zero-filling	14.57	26.1770	0.7744	18.41	24.1433	0.7102
	DIP	9.18	30.1892	0.9355	11.01	28.6124	0.9077
	Proposed method	**7.27**	**32.2166**	**0.9597**	**8.13**	**31.2440**	**0.9458**

**Table 5:** Relative errors, PSNR, and SSIM values of reconstruction for Brain B by different methods at 30% sampling rates under the radial undersampled mask.

Methods	Relative Error (%)	PSNR (dB)	SSIM
Zero-filling	11.28	29.3139	0.8694
DIP	8.61	31.6610	0.9339
Proposed method	**6.98**	**33.4717**	**0.9490**
